# Better together: Service user and delivery staff experiences of the SPACES physical activity intervention for people with severe mental illness - a qualitative study of a feasibility trial

**DOI:** 10.1016/j.mhpa.2025.100717

**Published:** 2025-10

**Authors:** Eleanor Bowes, Trys Burke, Matthew Faires, Gareth Jones, Kasia Machaczek, Helen Quirk, Gemma Traviss-Turner, Rebecca Beeken, Emily Peckham

**Affiliations:** a Institute of Health Sciences, University of Leeds, Leeds, UK; b School of Health Sciences, Bangor University, Bangor, UK; c Institute for Health and Care Improvement, York St John University, York, UK; d Advanced Wellbeing Research Centre, Sheffield Hallam University, Sheffield, S9 3TU, UK; e Sheffield Centre for Health and Related Research, School of Medicine and Population Health, University of Sheffield, UK

**Keywords:** Severe mental illness, Physical activity, Sedentary behaviour, Social support, Acceptability, Feasibility

## Abstract

**Background:**

Life expectancy is reduced by around 15–20 years for people with severe mental illness (SMI) compared to those without SMI, and this gap is widening. Increased physical activity is one way to reduce this mortality gap. The SPACES programme was designed to develop and test an intervention that supports people with SMI in increasing their physical activity. This paper presents the findings of a qualitative study conducted within the SPACES feasibility trial, which explores the experiences of both participants who received the SPACES intervention and those who delivered it.

**Methods:**

Qualitative semi-structured interviews were conducted with thirteen people with SMI who participated in the intervention, as well as six Physical Activity Coordinators (PACs) who delivered the intervention within the NHS. The interviews explored the participants’ perceptions of the intervention, including its feasibility, acceptability, potential benefits and insights into its implementation. The transcripts were analysed, and themes were generated using Framework analysis.

**Results:**

The SPACES physical activity intervention was perceived positively. Both PACs and people with SMI identified key enablers and barriers to implementation and continued engagement in physical activity. Key enablers were primarily social and included aspects such as social connectedness, personalised one-on-one support from professionals, and the welcoming and accepting atmosphere of community venues. Barriers included small group sizes, which did not lead to social connections, logistical challenges, inconvenient venue locations and travel issues. The PACs who delivered the intervention felt well-equipped to anticipate and address potential challenges in future intervention implementations.

**Conclusion:**

The co-designed SPACES physical activity intervention was acceptable to people with SMI and was considered feasible by PACS delivering the intervention. The results have wider implications for understanding how to achieve and maintain engagement in physical activity for this population. They further highlight the need for more holistic approaches to physical activity programmes tailored for people with SMI. Such approaches should emphasise social connectedness, foster welcoming community-based centres, and provide ongoing personalised support and guidance to help individuals with SMI integrate into community-based physical activity opportunities.

## 1 Background

People with severe mental illness (SMI), including schizophrenia, bipolar disorder, and schizoaffective disorder, face significantly higher health risks than the general population (Vancampfort et al., 2017). They are 3–5 times more likely to develop physical conditions such as heart disease and diabetes (Correll et al., 2017; Reilly et al., 2015; Wei et al., 2022), contributing to a reduced life expectancy of 15–25 years (Hayes et al., 2017; Laursen et al., 2019). Many of these deaths result from preventable conditions linked to inactivity, sedentary behaviour (Bull et al., 2020; Vancampfort et al., 2017) smoking and poor diet (Reilly et al., 2015). In the general population, physical activity (PA) and reduced sedentary behaviour (SB) are associated with lower risk of cardiovascular disease, diabetes, and improved mental health (Biswas et al., 2015; Stubbs et al., 2018).

People with SMI typically engage in less PA and more SB than the general population (Mishu et al., 2019; Vancampfort et al., 2017) due to specific barriers such as mental health symptoms, limited social support, and sedative medication side effects (Firth et al., 2016). Increasing PA in this group could help reduce the mortality gap. Beyond physical benefits, PA positively affects mental health, cognition, and brain function in people with schizophrenia (Rimes et al., 2015). However, evidence on effective PA or SB interventions remains inconsistent and low quality (Ashdown-Franks et al., 2020; Traviss-Turner et al., n.d.). Robust RCTs using objective PA/SB measures are needed to identify effective interventions (Chapman et al., 2024; Howes et al., 2024) however more research is required. Preliminary findings suggest that interventions should include motivational components and supervised sessions, with future studies testing longer-duration, higher-frequency programmes (Chapman et al., 2024). Co-development work with PPIE groups (Walker et al., 2023) indicates the value of community-based delivery, with a graded approach to sustaining PA. In the United Kingdom (UK), further research is needed to assess the feasibility and acceptability of embedding PA interventions into National Health Service (NHS) pathways (Jones et al., 2024).

No large-scale studies have yet evaluated implementation of sustainable PA interventions as part of routine care for this population. Understanding perspectives from both participants and delivery staff is therefore vital. Co-author Helen Quirk (HQ) has reported on the multistep process required to increase PA in people with SMI, including readiness for change, cost, safe spaces, and social support (Quirk et al., 2020). This aligns with recovery frameworks like CHIME, which emphasise connectedness, hope, identity, meaning, and empowerment (Leamy et al., 2011). The SPACES programme was developed to co-produce and evaluate a PA intervention embedded within NHS care, with a strong social and community focus, aimed at increasing and sustaining PA in people with SMI. It is important to note that there is no one agreed definition of SMI, therefore for the purposes of the SPACES study, and in consultation with the SPACES PPIE group, we have defined SMI as being people who would be eligible to be on the UK General Practice Quality Outcomes Framework SMI register and refer to the people who took part in our study as people with SMI.

### 1.1 Overview of the SPACES programme

**Fig. 1:**
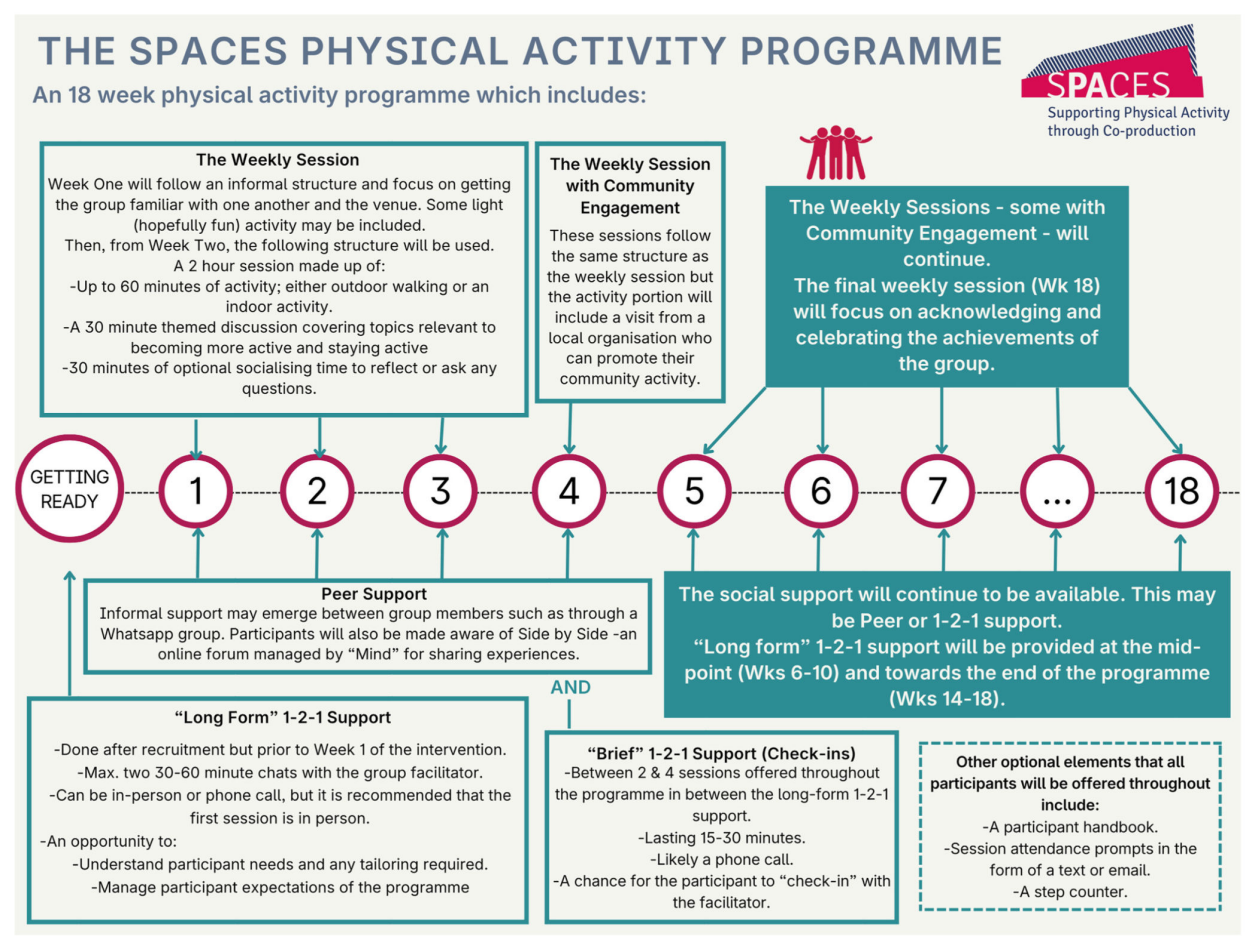
SPACES intervention infographic (Jones et al., 2024) Click or tap here to enter text.

The SPACES intervention was co-produced and tested in a feasibility trial (Jones et al., 2024) This paper describes the qualitative findings from the SPACES feasibility trial, with a particular focus on participant and provider experiences. The co-production process is reported separately (Walker et al., 2023). Fig. 1 provides an overview of the SPACES intervention delivered during the feasibility trial.

The SPACES intervention was an 18-week, group-based physical activity programme delivered across six NHS mental health trusts in England. Sessions were led by Physical Activity Coordinators (PACs), trained by the SPACES research team. Guided by the COM-B model and Behaviour Change Wheel (Michie et al., 2011), the intervention comprised of weekly group sessions (six walking, six indoor, and four delivered by local community providers) alongside 30 min of themed discussion and 30 min of social time. Participants were also offered one-to-one sessions with PACs to support motivation, goal setting, and address individual concerns. The underpinning theoretical approach of the SPACES intervention was the Behaviour Change Wheel, which poses the importance of Capability, Opportunity, and Motivation on Behaviour (COM-B) and acts as a hub for behaviour change (Michie et al., 2011). This model helped to guide the exploration of service users’ and PACs’ experience of the SPACES intervention and aided analysis in this study as a framework in which to reflect upon.

The feasibility study aimed to assess recruitment and retention for a future full-scale RCT and to refine trial procedures. These findings will be reported separately. To understand participants’ experiences, qualitative individual interviews were conducted, allowing deeper insight into how the intervention influenced behaviour change. Moreover, gaining perspective of both the service users and delivery staff enabled the research team to better understand what people living with SMI need in terms of capability, opportunity, and motivation to make changes in their behaviour toward a more active and less sedentary lifestyle. These insights also informed strategies to better support staff in facilitating those changes.

### 1.2 Objectives

The aim of this study was to explore the experiences of both those who received the SPACES intervention and those who delivered it with a view to improve participant capability, opportunity and motivation to make sustained changes in their behaviour toward a more active and less sedentary lifestyle.

## 2 Methods

The SPACES feasibility study was registered with the ISRCTN registry (ISRCTN83877229, registered September 9, 2022) and received ethical approval from the West of Scotland NHS Research Ethics Committee (ref: 22/WS/0101, approved September 1, 2022). Data collection, storage, and processing complied with the Data Protection Act 1998. A risk protocol was in place for disclosures of serious harm. The research team has extensive experience working with people with SMI, and interviewers were trained in assessing capacity.

### 2.1 Participants

Service users who received the SPACES intervention and the PACs who delivered it were invited to take part in one-to-one interviews. Purposive, heterogeneous sampling was used to capture a range of engagement levels and demographic characteristics, including age, gender, prior physical activity, session attendance, and ethnicity. Typically, 2–3 service users per site were contacted. Interested individuals were approached via email and/or phone, with one reminder sent. Of 20 service users contacted, 13 consented to participate. PACs were invited based on availability, with 1–2 per site selected after completing their delivery of the intervention. All participants provided informed consent for participation and audio recording (see Supplementary File 1). Interviews were conducted in person, by phone, or via Microsoft Teams, depending on participant preference, and recorded using encrypted digital recorders. Interviews averaged 28 min. Recordings were transcribed by a professional service, and all identifiable information was removed. Participants received a £20 voucher as thanks for their time.

### 2.2 Data collection

Conduct of the interviews and the reporting of the findings was guided by the Consolidated Criteria for Reporting Qualitative Research (COREQ) (Tong et al., 2007), which is a 32-item checklist for interviews and focus groups (see Supplementary File 2).

EB, MF, GJ, EP, HQ, and LB (see acknowledgements) developed two topic guides: one for service users and another for PACs (see Supplementary File 3). To develop the guides, the researchers met to discuss what information they wanted to gather from the interviews. Questions were subsequently drafted and shared with the wider group for suggestions and comments. Following this, the draft topic guides were shared with the SPACES PPIE group for comment on the acceptability and language of the questions. MF and EB guided service users and PACs through the topic guide. The service user’s topic guide covered the following areas of experiences:

- Accessibility to the intervention
- Acceptability of the intervention
- Reasons for dropping out, if applicable, and alternatives needed
- Potential benefits of the intervention.

The topic guide for PACs explored their experiences of:

- Training, ongoing support and delivery resources provided
- Confidence and transferrable skills found useful
- Feasibility of the intervention including difficulties experienced during delivery and improvements that could be made.
- Aspects they considered service users felt most valuable

Data collection was finalised once the research team believed data saturation had been reached, defined by: A) no new themes or codes: after analysing several interviews or data units, the research team noticed that no new themes, patterns, or codes were emerging and B) repetition of information: participants’ responses start to become repetitive, and additional data largely confirm what has already been found rather than adding fresh perspectives.

### 2.3 Analysis

Transcripts were initially read independently by two authors (EB and TB) using thematic reading and analysed using framework analysis (Goldsmith, 2021). Framework analysis aims to identify, describe, and interpret key patterns within and across cases and themes within the phenomenon of interest following five steps (Goldsmith, 2021; Parkinson et al., 2016; Ritchie & Spencer, 2002). Table 1 summarises the research team’s actions to achieve each step. Further, thematic development of the themes was guided by Braun and Clarke (2021) and Nowell et al. (2017).

## 3 Results

### 3.1 Sample

Thirteen service users and six PACs participated in interviews conducted between August and October 2023 (see Table 2). A heterogenous sample was recruited with a range of ages, diagnoses and ethnicities. Service users were recruited from five of the six participating NHS sites in England. Five of the six PACs were female; all except one had training (diploma and/or degree) or experience in facilitating physical activity, and all had experience working with people with SMI. Service users with a range of group physical activity session attendance rates were interviewed. Participants who received 0 % attendance at the intervention provided a valuable perspective, as they met inclusion criteria and were somewhat motivated to participate in the study, however, at some point between randomisation into the intervention group and the intervention starting, either circumstances or motivation changed. These service users still had access to the participant handbooks, and attended at least one 1:1 appointment with their PAC.

### 3.2 Themes identified from service user and PAC experiences

The experiences of service users and PACs during the SPACES feasibility trial is initially explained via 4 themes: 1) social support, 2) variety, structure, and permission to play, 3) venue choice, and 4) factors aiding the PAC’s facilitative role for behaviour changes in service users (see Table 3). We also present the themes and sub-themes through the lens of the COM-B model to highlight the factors found in this study as influencing service user’s physical activity behaviour.

#### 3.2.1 Theme 1: “Coming together” - social support

In the first theme, service users and PACs emphasised the significance of social interaction with peers, PACs, and community groups for the success of the intervention and their ongoing participation. Within this broader theme of social support, several subthemes were identified including shared experiences, safe social space, facilitated group exercise, shared knowledge, and small but viable group size.

#### 3.2.1 Shared experience

SPACES provided service users with opportunities to engage in group activities with others who shared similar SMI experiences. PACs observed a noticeable “*progress in the relationships that people developed within the group*,*” (PAC 3)* and service users spoke of how they enjoyed meeting others with lived experience of mental illness.

“You don’t get much opportunity to talk to people about your mental health experiences […] So to actually have that opportunity to come together with people” (Participant 8).

##### 3.2.1.2 Safe space

Drawing from the sub-theme above, participants reported that the social time structured the session and provided them with a safe social space for shared experience. Having a safe physical space was also important. Having others who had lived experience or understanding of living with SMI created a safe space for socialising:

“It was nice for him [other service user] I think, to feel like he had someone else he could talk to […] to feel it was safe to talk to somebody.” (Participant 8).

Programme accessibility was also heavily influenced by venue characteristics, such as a safe and welcoming space. For example, one participant mentioned they preferred *“a venue in a familiar location”* (Participant 9). PACs too reported on welcoming atmosphere of a venue and its benefit to the service users,

*“Very accessible, people were understanding of additional needs, it felt very welcoming”* (PAC 3)

Social interaction that took place within the intervention was identified as a major motivational factor, contributing to both acceptance and accessibility of the SPACES intervention being able to get to the venue easily was identified as critical in facilitating this. Both service users and PACs identified ease of travel to and from the venue (i.e., a single use of public transport) as being essential to accessing the intervention. Participants stated that being able to take a single reliable form of transport rather than changing was preferable.

“Sometimes I just managed to get a bus that took me straight through… what would have helped me would have been a taxi there” (Participant 1)

One PAC reported that their participants liked the venue, but that it was *“just a bit too far away from all of them”* (PAC 3). Many experiences within this theme reflect the dichotomous nature of venue choice. For example, when PAC 3 asked if a city centre venue would have been better the participants said no, “*because most found the city centre a bit overwhelming*”. In complete contrast Participant 1 suggested a more *“central location might be better”*. Dichotomousness was present within the PAC experiences too. Although recognised as a critical aspect of the intervention, some PACs considered *“the perfect venue doesn’t exist”* (PAC 1); whilst others who had managed to find the ideal venue stated it to be *“a perfect venue and a perfect location”* (PAC 7).

##### 3.2.1.3 Facilitated group exercise

Participants identified that being part of a group as a key motivator for attendance and staying active.

“It was more encouraging to be there as a few people in a team” (Participant 1);

“I think a lot of it was the social aspect as well and it actually got me to doing more exercise” (Participant 4).

Consistent with participant experiences, PACs suggested that building social networks facilitated physical activity and improved the service users’ motivation and confidence:

“You’ve got somebody who wasn’t communicative at all and didn’t speak and just came and did what he had to do. But then as the weeks were going on, you could see that they were becoming more confident even if they didn’t communicate verbally a lot” (PAC 6).

##### 3.2.1.4 Shared knowledge

Themed discussions facilitated social connection and helped with capability by “*sharing and expanding our awareness around certain topics.” (Participant 6)*. This enabled service users to pool their knowledge and share strategies to improve physical activity.

“It wasn’t just about the exercise that it was just about that social support and the communication between us over that 18 week and the talking, understanding, listening, making suggestions of things that you might be able to change to make things better” PAC 6).

##### 3.2.1.5 Small but viable group size

Group size also appears to be a critical factor influencing motivation and continued engagement with the intervention. Overall PACs believed service users preferred smaller groups with a closer service user to PAC ratio.

“With it being smaller, they [service users] could really just engage one-to-one with you in the group. […] But actually it being a smaller group, it made it a bit easier.” (Participant 12).

That said, service users felt that if numbers dropped to a point, it would negatively affect motivation and engagement and inhibit any developments in socialisation and activity variability. One service user suggested,

“It got a bit boring because no one was turning up and […] we were just walking and stuff” (Participant 5).

Another service user stated that they were happy but that,

“it might have been a little bit better if there would have been more [people].” (Participant 1)

Increasing the minimum group size to 8 was deemed necessary for the group to be viable and successful, needing to account for drop out and low attendance.

“I think you need to over recruit. I think that’s evident” (Participant 8)

#### 3.2.2 Theme 2: Variety, structure, and permission to play

This theme focuses on how the structure and content of sessions impacted service users’ acceptance of, and motivation to remain engaged in the SPACES intervention. “Permission to Play” was identified as a key theme from PAC feedback. This phrase captures the sense of freedom, acceptance, and emotional safety that PACs observed individuals with severe mental illness experiencing during the intervention’s warm-up games. These playful activities represented a rare opportunity to engage in physical activity without judgment or performance pressure—offering not just movement, but moments of joy, connection, and self-expression.

##### 3.2.2.1 Intervention duration supported change

A 2-h intervention over 18 weeks supported behaviour change by allowing sufficient time for developing confidence and forming physical activity habits. Service users reported that conducting SPACES over 18 weeks, rather than a shorter duration, was “key” *(Participant 7)*.

“ I think the 18 weeks was perfect. It’s just enough to get you sort of like more comfortable and then you can go out and do something yourself “ (Participant 12)

Although some service users felt slightly shorter sessions *(“I think an hour and a half” - Participant 8)* would suit them better, others felt that the 2 h provided a buffer for late arrivals:

“I don’t think you could reduce it much more” (Participant 1)

##### 3.2.2.2 Varied intervention components

The varied intervention components appeared to support engagement. PACs reported how different elements of the intervention motivated people in different ways,

“[Service users] enjoyed being more involved with planning activities as time went on” (PAC 2); “[They] really enjoyed the warmup games [dodgeball] it gave them permission to play” (PAC 3).

Importantly keeping things simple by repeating some elements appeared to be a key factor to increasing confidence as *“familiarity and the repetition seemed reassuring” (PAC 2)*.

The service users highlighted the importance of having a varied programme and being shown ways to be physically active in a non-exercise way, such as walking to a further bus stop. Different components of the SPACES programme motivated service users and fostered their psychological capability (e.g., confidence) to be more physically active and less sedentary.

Community-based sessions and outdoor walks supported capability and motivation by bringing awareness of available classes and clubs and providing the chance to try the sessions in a safe space, facilitating post-intervention uptake.

“Everybody loved it [the yoga class] and they were even talking about, you know, signing up to a class with her.” (Participant 4).

Walking sessions highlighted routes service users “*didn’t know existed*” *(Participant 1)*, facilitating increased walking post-intervention. Similarly, the indoor activities introduced service users to new ways of being active such as using exercise bands with options offered that were “*simpler than I thought it would be” (Participant 3)*.

Finally, the mindfulness element was well-received by service users and became a helpful addition to skills developed during the SPACES intervention.

“I learnt mindfulness practices that have become a part of my daily life without necessarily trying so much” (Participant 8).

The above sub themes suggest that as capability increased, motivation increased, too. Nevertheless, although many responses were positive, some service users identified reasons that reduced their motivation. For instance, some service users found the themed discussions repetitive and overly simplistic, hindering their engagement.

“I didn’t like that aspect of it [the intervention] at all. It was the same conversation that was being had over and over again.” (Participant 8).

Overall, however, SPACES was an acceptable intervention for service users, facilitating motivation to be more active and overall confidence in new activities, even outside of exercise.

“Since SPACES, because I’m getting a bus there and back every week, I’ve got a lot more confidence with it. So, I’m looking to go into voluntary work. And then if I can’t cope, it doesn’t matter, does it?” (Participant 12).

##### 3.2.2.3 Tailoring content to service users’ abilities

Tailoring content to service users’ abilities supported inclusion. PACs identified the need to tailor activities to service users’ baseline mobility and coordination.

“[Service users’] severe lack of mobility and coordination at start - I think the biggest challenge was probably the fact that none of them were moving very much beforehand” (PAC 5).

PACs found they had to simplify tasks into more steps than anticipated *“to make it really clear what we were asking them to do.” (PAC 5)*

Overall, PACs found similar themes in delivering physical activity interventions to people with SMI and to people without SMI, such as managing expectations and maintaining a balance between structure and flexibility.

#### 3.2.3 Theme 3: Factors aiding PACs’ facilitative role

This theme explores key factors that PACs identified as supporting their ability to motivate and sustain service user engagement in physical activity. These included taking a non-illness-focus, session structure and content (discussed previously in Theme 2 above), training and supervision, and the benefit of transferable and shared skill sets among PAC teams.

##### 3.2.3.1 Not illness focused

The training PACs had received involved two days, the first focusing on ‘how’ to deliver the intervention and the second on ‘what’ to deliver. The ‘how’ aspect focused on mental health training, including how best to communicate and facilitate the service users’ capacity, opportunity, and motivation rather than emphasising physical activity qualification. The training was soft skill focused rather than hard skill focused, and the PACs reported that the intervention’s non-illness approach enabled them to foster more inclusive and empowering environments. Shifting the emphasis from medical diagnoses to service users’ strengths, enjoyment, personal goals, and community involvement appeared to support greater engagement and longer-term participation in physical activity:

*“It [the SPACES intervention] wasn’t illness focused, people didn’t feel like they were coming to treatment, they were attending a group which was therapeutic, got them out, got them in the community, got them socialising.”* (SO6)

This approach was seen as especially valuable for this population, for whom traditional clinical settings may carry stigma or negative associations. By focusing on autonomy, enjoyment, and social connection, PACs were able to engage service users in a more positive and holistic manner.

##### 3.2.3.2 PAC training, supervision, and support

PACs described the initial training, resources, and ongoing supervision as critical in building confidence and competence. The two-day training course and PAC manual were well received:

“The training and resources were really comprehensive.” (PAC 2); “Sessions on motivational interviewing, mindfulness training, behaviour change – I found really useful.” (PAC 2); “The theory was spot on.” (PAC 1)

Training was generally viewed as enjoyable, although several PACs suggested improvements, including more practical demonstrations and clearer examples of physical activity delivery:

“Wished the training was in two halves: 1) the processes in terms of collecting information, 2) and a practical ‘this is what it could look like.’” (PAC 3)

The PAC manual was also seen as a valuable resource, offering clear guidance that could be adapted using the PACs’ own judgement and expertise. However, some expressed the need for more exercise examples, particularly for those with less experience in delivering physical activity interventions:

“Those types of exercises would have been hard [to deliver without prior experience].” (PAC 6)

Ongoing supervision and peer support were also seen as essential. Monthly online support sessions provided a space to share experiences and troubleshoot challenges. When attendance was not possible due to work commitments, PAC teams often rotated attendance and shared learnings:

“Helpful to share experiences of delivery and talk about any issues.” (PAC 4)

##### 3.2.3.3 Transferrable and shared skills set

Working in teams with complementary skill sets was seen as a major enabler of effective delivery. PACs who brought experience in physical activity, occupational therapy, or nursing tended to report greater confidence. The ability to share responsibilities and draw on diverse professional and personal experiences, including lived experience of SMI, was particularly valued:

“The most valuable thing for me was two PACs who’d both got lived experience and used exercise as a coping mechanism […] they connected really well with everybody.” (PAC 6)

Team-based delivery and shared responsibilities not only helped manage workload but also fostered a supportive environment for both PACs and participants.

### 3.3 Factors explaining physical activity behaviour based on the COM-B model

**Fig. 2:**
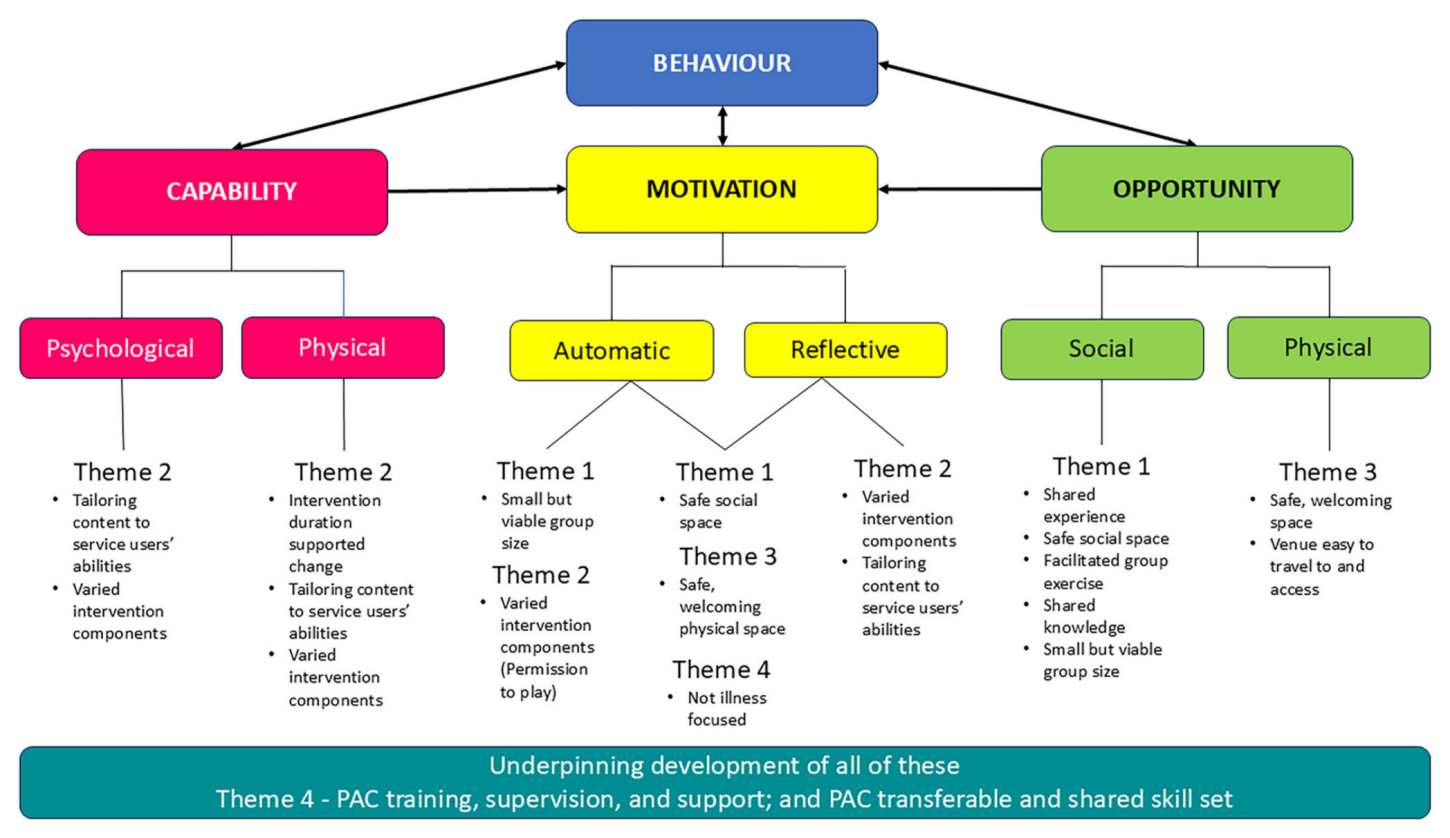
Factors identified as facilitating physical activity in people with SMI as seen through the lens of the COM-B model.

The subthemes identified from the data analysis were later mapped to the COM-B model to identify which factors from this study explained the physical activity behaviour of those taking part in the SPACES feasibility trial (see Fig. 2). This mapping exercise revealed that the factors identified by service users and PACs as facilitating and/or hindering physical activity touched on all components of the COM-B model.

#### 3.3.1 Capability

Capability refers to an individual’s psychological and physical capacity to engage in an activity (Michie et al., 2011). Three subthemes under this COM-B component (varied intervention components, tailoring to ability, and intervention duration) help explain service user engagement. Psychologically, participants were challenged through discussions, social interactions, and one-to-one sessions. Physically, they built capacity through gradual exposure to activities that respected their limits. The 18-week duration, longer than typical 6–12 week programmes, allowed time for growth, confidence-building, and what many described as a feeling of ‘permission to play’. PACs’ sensitivity to individual needs further supported engagement.

#### 3.3.2 Opportunity

Opportunity includes external factors -physical and social-that enable or prompt behaviour (Michie et al., 2011). Two key themes emerged: social support and venue choice. Social connection played a major role in behaviour change, with participants valuing the shared lived experience of their peers. The group setting created a safe space where service users felt free to be themselves. A supportive physical environment was equally important, offering a secure place to connect and be active.

#### 3.3.3 Motivation

Motivation involves the internal processes—both reflective and automatic—that energise and direct behaviour (Michie et al., 2011). Six subthemes were identified: varied components, tailored content, non-illness focus, permission to play, safe social space, and welcoming physical space. Elements such as playfulness, non-clinical framing, and supportive settings triggered automatic motivation through emotion and associative learning. One-to-one sessions and tailored activities helped service users reflect on these experiences, deepening self-awareness and building sustained motivation by encouraging them to plan for continuation of these positive behaviours.

Underpinning the development of all the COM-B elements was the PACs’ training, supervision, and support; and their transferrable and shared skill set.

## 4 Discussion

This study explored the experiences of receiving and delivering the SPACES intervention. While previous studies have examined feasibility and acceptability, few have investigated how such interventions influence participants’ capability, opportunity, and motivation for long-term behaviour change toward increased activity and reduced sedentary behaviour.

Findings showed that service user engagement was shaped by social support, a varied and structured programme, and a safe, welcoming environment. Delivery by trained staff who offered individualised, non-illness-focused support helped create a more empowering and motivating experience.

These results build on earlier work (Quirk et al., 2020) describing the gradual process of physical activity uptake for people with SMI. Social connection emerged as a key driver, alongside practical elements like group size, venue, and location.

***Social*** support ***(theme 1):*** People with SMI often have limited community contact and engage in fewer out-of-home activities (Stain et al., 2012). Service users and PACs emphasised the role of social support and group cohesion in SPACES’ success. Safe, comfortable spaces enabled open discussion of experiences with SMI and physical activity. Regular sessions with consistent group members over 18 weeks boosted confidence and motivation, with some continuing to meet post-intervention. This finding corroborates those of Lesley and Livingwood (2015), who found that trust-based relationships support physical activity engagement. Social connection was also a motivator for joining SPACES, highlighting its relevance for both motivation and capability.

Group size was also critical. Low attendance discouraged engagement, while very large groups felt intimidating, concerns echoed during the co-production phase. An optimal group size appeared to be 6–12, ensuring a critical mass while maintaining comfort. A minimum of six attendees is recommended, rather than six as an average, to allow for absence due to illness or other issues. Attendance varied considerably, suggesting recruitment strategies should account for this fluctuation while maintaining group cohesion. Larger groups may also enrich themed discussions by offering more diverse perspectives.

Many service users valued the themed discussions for encouraging idea sharing and informal conversation, enhancing both session structure and social interaction. However, some found the content repetitive or overly simplified. Further stakeholder consultation is needed to refine the themes and improve their relevance and appeal.

Service users identified easily reaching the venue by bus or similar public transport as highly important. Initially, focusing on a venue for what it offered the intervention, e.g., appealing walking routes became an issue when service users could not get there easily. Transportation issues for engagement in the community have been seen in other studies working with people with SMI (Pahwa et al., 2022; Slanzi et al., 2023) and other populations, such as those over 65+ and dependant on public transport (Sansano-Nadal et al., 2019). Venues outside of city centres were most likely to present challenges to service users, particularly when a service user lived in a different part of the city. A recent community participation study for individuals with SMI in rural areas (Slanzi et al., 2023) identify community mobility and access to a good public transportation network as key determinants of activity engagement. Location should therefore be considered to support capability and opportunity of service users.

***Variety, structure, and permission to play (theme 2):*** Providing activity personalisation through different activity options with tailoring for an appropriate entry point was important for enjoyment and engagement with physical activity. This finding concurs with other studies who stress the importance of personalising physical activity programmes for this population (Beckers et al., 2023; Machaczek et al., 2022; Soundy et al., 2012). Machaczek and colleagues (2022) suggested personalised programmes that incorporate a person’s wider physical and social context to better support people to adopt a more physically active lifestyle. Moreover, providing a range of activity types allowed service users to find forms of physical activity they enjoyed and found meaningfulness. This increased their motivation to continue participating in these activities even after the intervention finished. The options included local classes led by the instructors from community sessions and self-organised exercises like walking or at-home workouts. Accessibility and availability has previously been reported as a key barrier to physical activity (Heinz et al., 2025), therefore community sessions and PAC support may help to overcome this barrier. Another SMI study (Farholm et al., 2017) has found similar results and stress that by offering relevant choices, supporting patients’ initiatives, providing meaningful rationales and avoiding extensive external rewards, practitioners can foster greater autonomous motivation necessary to maintaining a physically active lifestyle. Whilst it was found that it was feasible to create a varied but structured programme, it is possible that with a different group composition it might not be possible to create a programme that suited everyone.

Tailoring the intensity and difficulty of activities offered also allowed PACs to offer SPACES at a suitable level for different service users. This was essential from a safety perspective, reducing injury risk, but also supported service users’ capability and engagement of the intervention.

Within the SPACES programme, PACs reported gradually increasing their service users’ choices and decision-making opportunities aiming to make activities more sustainable after the intervention. The sustainability and maintenance of activity after the SPACES intervention ends will be tested further in the main SPACES trial, with data collection occurring at 12 months. Social cohesion and activity tailoring may help to overcome the challenge of maintaining the health-related benefits acquired by the intervention’s recipients once it ends (Weber et al., 2018). In their systematic review of physical activity interventions for older (65+) adults, Sansano-Nadal and colleagues (2019) found that supervised exercise interventions with specific strategies to enhance sustainability had statistically significant beneficial effects on physical activity levels at the end of intervention compared to non-active controls or active controls. One study in particular included key mechanisms to enhance social support during the cool-down phase of each session, such as social influence/social comparison, social control, self-esteem, sense of control and belonging, and companionship (Martín-Borràs et al., 2018).

**Factors aiding the PACs’ facilitative role (theme 3):** PACs identified creating a non-illness focus was highly beneficial to the service users. Adopting a non-illness approach has been reported to restore mastery and control to patient’s life in those suffering with a disability (Navon, 2005). Empowerment is a vital element for behaviour change, Hsieh et al. (2023) in their systematic review found empowerment in illness management can effectively promote medication adherence and recovery of persons with schizophrenia using strategies that were not illness focused but instead focused on understanding personal needs and overcoming self-stigma.

The PACs further identified their training as being invaluable in preparing them for the intervention and the challenges they would meet. Our findings suggest the role of the PAC in facilitating capability, opportunity, and motivation was vital to the success of the intervention, moreover ensuring they are trained and equipped with the ability to ‘step back’ at an appropriate point in time is essential if the intervention group is to be able to maintain their behaviour change once the trainer/delivery staff is no longer with them. This notion is support by literature into peer support interventions. Christensen et al. (2021) stress the importance of equipping experienced peers and (paraprofessional trainers) to orient less experienced peers to their roles in facilitating ongoing and sustained interactions, and to fade their proximity over time. Peer support training and train-the-trainer (TTT) programs are frequently used to facilitate interventions and dissemination of knowledge; however, little is known about the effectiveness of these TTT programmes (Poitras et al., 2021). Following their systematic review, Poitras et al. (2021), found the heterogeneity and small number of studies available studies hampered their ability to draw conclusions that such programmes were indeed effective. More research into the effectiveness of TTT programmes is necessary especially in terms of COM-B and will be considered further in the SPACES full trial.

### 4.1 Bringing it all together

The multi-component SPACES intervention for improving physical activity in people living with SMI was acceptable to both service users and PACs. No single aspect of the intervention was responsible for making it acceptable; instead, it was the combination of social connectivity, appropriate content, tailoring, accessible venues and suitable PAC training and support. The benefit of SPACES comes from the ‘whole’ being greater than the ‘sum of its individual parts’. Relating the results to the COM-B model, findings reinforce the importance of both social and environmental opportunity in that the intervention was both accessible practically, but also enabled social interaction, and was appropriately pitched and facilitated by PACs to aid psychological and physical capability (Michie et al., 2011). Psychological capability was especially important for early experiences of the SPACES intervention to build service user confidence. These aspects then had an impact on service user motivation, as initial desires to be involved in SPACES (e.g., social connection) were being met.

Regarding the journey to living an active life, the SPACES approach enabled people living with SMI who have begun their journey to initiate physical activity (Quirk et al., 2020; Soundy et al., 2012), maintain it. Quirk and colleagues (Quirk et al., 2020) identified three themes relating to taking part in physical activity for the first time: socialisation and the influence of the group, accessibility and scheduling, and immediate benefits of taking part. Service users in the SPACES feasibility trial also saw the benefit of social time and the need for the location to be accessible.

The experiences of our service users and PACs provides additional insight into ways of continuing the journey and maintaining activity. This concept is encapsulated in the phrase “better together”. The experiences of our service users suggest that continued input from trusted people, continued socialisation with peers, continued increase in confidence, trusted person to continue building autonomy. All of these will maintain the individual’s motivation and thus their engagement in physical activity.

### 4.2 Strengths and limitations

A strength of the current study was the diversity of the sample in terms of background characteristics and geographical spread across UK NHS sites. The findings add important contextual detail to the feasibility study outcomes. Limitations included service users being recruited post 6-month follow-up, meaning no interviews were conducted with people who declined to participate in the intervention. Including these perspectives would have provided greater understanding of perceptions of the intervention.

### 4.3 Recommendations for practice

This study was part of the feasibility study thus recommendations are designed to inform the full trial after which more nuanced recommendations will be forthcoming. This paper demonstrates the importance of social connectiveness to increase the acceptability and success of the SPACES intervention. Including ice breaking activities, structured and unstructured social/discussion sessions and maintaining consistent groups may support social connection in similar interventions. In terms of venue choice, balancing the need for a safe, welcoming venue in a pleasant area with a location that is accessible to expected participant home addresses is key. Finally, appropriate training and ongoing support for PACs is essential for empowering PACs to lead sessions as intended and with the ability to adapt to meet participant needs.

## 5 Conclusion

The co-designed physical activity intervention, SPACES, was acceptable to service users with SMI and deemed feasible by the NHS staff who delivered the intervention. The results have wider implications for understanding how to achieve increased engagement in physical activity for this high-risk population and further underscore the need for more holistic approaches to physical activity programming for people with SMI. This holistic approach includes a greater emphasis on social connections, welcoming community-based centres, and continued personalised support and guidance, from a trusted professional, to help integrate those with SMI into community-based physical activity opportunities, thereby ensuring engagement in physical activity is sustained for longer.

## Supplementary Material

Supplementary data to this article can be found online at https://doi.org/10.1016/j.mhpa.2025.100717.

Supplementary Material

## Figures and Tables

**Table 1:** Actions taken by the research team to achieve each step of framework analysis.

Step	Actions taken by the research team
***Step 1. Data familiarisation***	EB and TB independently reviewed all the service user and PAC transcripts, with EB focusing on service user transcripts and TB focusing on PAC. They identified key ideas related to participants’ experiences.
***Step 2: Framework Identification***	EB and TB discussed the data, a priori codes derived from components of the COM-B model, the topic guide, and potential themes identified from the initial familiarisation exercise above enabled a coding framework to be developed.
***3: Indexing***	EB and TB systematically applied the framework to all the study data. They initially organised the data using two units of analysis: service user experience and PAC experience. The data was combined when the readings indicated consensus in some core themes across both data sets. This process was iterative; themes and subthemes were merged, split and renamed as necessary.
***4: Charting***	EB and TB summarised the data in a matrix by transferring quotes and service user/PAC ID numbers into a Microsoft EXCEL spreadsheet. Continuous cross-references to the original transcripts ensured that the information represented in the matrix was a true representation of the data set collected.
***5: Mapping and interpretation***	EB and TB created a coding tree (see Supplementary File 4) using Miro’s software “Brainwriting” function. The initial framework and emergent themes were independently reviewed and discussed with a third author to ensure consistency and credibility. During this verification process, discrepancies in coding or theme interpretation were identified and resolved through discussion, leading to consensus. As a result, several themes were refined for clarity, and minor modifications were made to the framework to better capture the nuances in the data. This collaborative review enhanced the trustworthiness of the analysis.

**Table 2:** Participant demographics.

User ID	Age	Sex	Ethnicity	Diagnosis^a^	Attendance of group sessions (%)	Drop out**
Participant 1	56	F	White British	F30 + F31	67	No
Participant 2	26	M	African	F21-F24	100	No
Participant 3	47	M	White British	F20	67	No
Participant 4	64	F	White British	F30 + F31	56	No
Participant 5	20	M	White Irish	F20	67	No
Participant 6	44	F	White and Black Caribbean	F20	22	No
Participant 7	38	M	White British	F20	22	WFT
Participant 8	44	F	White British	F30 + F31	44	No
Participant 9	32	F	White and Black Caribbean	F30 + F31	0	WFT before week 1
Participant 10	54	M	Black African	F25	0	WFT
Participant 11	50	F	Caribbean	F20	72	No
Participant 12	41	F	White British	F20	100	No
Participant 13	42	M	White British	F20	100	No
**PAC ID**	**Sex**			**Profession**		
PAC 1	M			Occupational Therapist		
PAC 2	F			Occupational Therapist		
PAC 3	F			Mental Health Nurse		
PAC 4	F			Research Assistant		
PAC 5	F			Psychologist		
PAC 6	F			Health and Exercise Practitioner		

a KEY: Diagnosis F codes are as follows: F20 – Schizophrenia, F25 – schizoaffective disorder, F21-F24 Other psychotic and delusional disorders, F30 + F31 - Bipolar disorder and manic episodes, WFT – Withdrawn from treatment.

**Table 3:** Themes identified.

Theme	Sub-themes
1. Social support	1.1 Shared experience
	1.2 Safe space
	1.3 Facilitated group exercise
	1.4 shared knowledge
	1.5 Small but viable group size
2. Variety, structure and permission to play	2.1 Intervention duration supported change
	2.2 Varied intervention components
	2.3 Tailoring content to service users’ abilities
3. Factors aiding PAC’s facilitative role	34.1 not illness focused
	34.2 PAC training, supervision, and support
	34.3 Transferable and shared skill set

## Data Availability

The authors do not have permission to share data.
