# Supplementary material for: Better together: Service user and delivery staff experiences of the SPACES physical activity intervention for people with severe mental illness - a qualitative study of a feasibility trial
Source: Ment Health Phys Act. Author manuscript; Available in PMC 2025 Dec 4. (PMC7618440; doi:10.1016/j.mhpa.2025.100717)
Supplement: Supplementary Material [file EMS210840-supplement-Supplementary_Material.zip › 1-s2.0-S1755296625000481-mmc1.docx]

**S**upporting **P**hysical **A**ctivity through **C**o-production in p**e**ople with **S**evere Mental Illness (SPACES)

**Consent Form - Interview**

**Part 1 (Main Consent)**

|  |  | **Researcher Initials** |
| --- | --- | --- |
| 1. I have read and understand the information sheet (enter version and date), have discussed the study with a SPACES team member and have had the chance to ask any questions 2. I understand that I will be interviewed about my experiences of the SPACES physical activity programme. I understand that I will not be identified by name but by a unique study code instead. 3. I consent to the feedback session being audio recorded. 4. I agree that researchers may use my anonymised data in publications, reports, web pages and other research outputs. 5. I understand that other researchers outside of the SPACES team can have access to my anonymised data for research purposes only. 6. I understand that my participation is voluntary and that I am free to withdraw at any time without giving any reason, and without my medical care or legal rights being affected. I understand that I need to let the study team know if I wish to withdraw from the study. 7. I agree to The University of York holding my contact details in order to contact me about the study. I understand that my contact details will not be shared with anyone outside the research team. 8. By signing this form (below) I consent to taking part in this SPACES interview given all the information I have received so far. | \|  \|  \| \| \| --- \| --- \| --- \| \| **Yes** \| \| **No** \| \| \| **Yes** \| \| **No** \| \| \|  \| \|  \| \| \| **Yes**  **Yes** \| \| **No**  **No** \| \| \| **Yes** \| \| **No** \| \| \| **Yes** \| \| **No** \| \| \| **Yes** \| \| **No** \| \| \| **Yes** \| \| **No** \| \| |  |

**Contact Details (please complete in BLOCK CAPITALS)**

First name:

Surname:

Address:

Postcode:

Phone number:

Email address:

**Participant Verbal Consent Confirmation**

**After explaining the SPACES study to the below named participant, I can confirm that the participant provided their verbal consent to take part in the SPACES study (as recorded above).**

**Full Name of Participant:**

**Date participant provided verbal consent to participate (dd/mm/yyyy):**

**Name of SPACES researcher taking verbal consent:**

**Signature of SPACES researcher taking verbal consent:**

**SPACES researcher Site ID:**

Office Use

Participant ID: _________ Consent Taken By: _______________________________________

Date: ________________

Copy of consent form (tick):

☐ Research File ☐ Participant ☐ GP
