# Supplementary material for: Better together: Service user and delivery staff experiences of the SPACES physical activity intervention for people with severe mental illness - a qualitative study of a feasibility trial
Source: Ment Health Phys Act. Author manuscript; Available in PMC 2025 Dec 4. (PMC7618440; doi:10.1016/j.mhpa.2025.100717)
Supplement: Supplementary Material [file EMS210840-supplement-Supplementary_Material.zip › 1-s2.0-S1755296625000481-mmc3.docx]

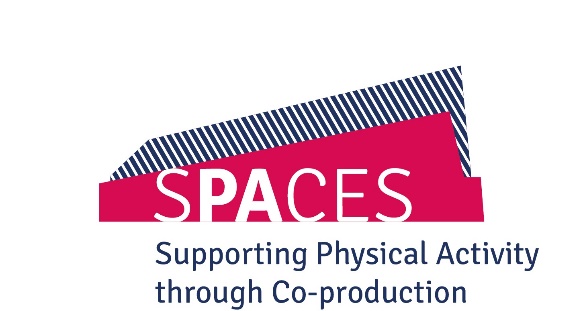


**S**upporting **P**hysical **A**ctivity through **C**o-production in p**e**ople with **S**evere Mental Illness (SPACES)

**Semi Structured Interviews**

Topic Guide for participants in the SPACES intervention arm

*Suggested questions are for guidance only. Use a semi-structured approach to explore ideas and views raised by participants where appropriate.*

**Introduction:** **Welcome and reminder of key points**

- Our interest is in your experiences of taking part in the SPACES intervention, you are welcome to share anything you would like about this.
- There are no right or wrong answers – we are looking to improve the intervention so all feedback, good and bad, is very valuable for us.
- Everything shared will remain confidential. The only time we may have to tell someone what you have said to us is if you say something which suggests there is a risk of significant harm to someone (including you).
- The conversation will be audio recorded but, once typed up, your responses will be anonymous and you will not be identified by name in any written reports. If participant happy to proceed turn on audio-recorder.

**What did you think about the SPACES intervention?**

*Optional prompts:*

**Accessibility:**

- How did you first hear about the SPACES intervention and what made you want to take part?
- Can you tell me about anything that helped or hindered your ability to attend the SPACES sessions?
- Information content - Was there anything that you particularly liked or disliked about the weekly workshops (themed discussions)?
- Was there anything that you particularly liked or disliked about the Participant Handbook that you were given?
- Frequency – How did you feel about the number of sessions you were offered?
  - *Do you think you were offered enough/too many one-to-one appointments?*
  - *Or would you have liked more/less? Can you tell us why?*
- Timing/Duration – How did you feel about the length of the sessions?
  - *Do you think that the weekly sessions were delivered over the correct time period, or would you have preferred them to be longer or shorter?*
  - *Do you think that the weekly sessions were delivered at an appropriate time of day?*
  - *How did you find fitting the intervention in around your other commitments?*
- Location – Can you tell me your thoughts about where the sessions took place?
  - *Do you think the venue for the weekly session was the best place for it to be delivered?*
  - *How would you describe your journey to the venue?*
  - *Did it cost much money for you to travel to the venue?*
  - *Did you need any support in travelling to the intervention? (a lift, someone to go with, a bus pass)*

**Acceptability:**

- What did you like about the intervention?
- Was there anything you disliked about the intervention?
- Did you find it easy or challenging?
  - In what way?
- Did you use any of the optional items? If yes, were they useful? If no, why not?
  - *session prompts*
  - *pedometer*
  - *handbook (layout, content)*
- Can you tell me how you felt about the physical activity part of the SPACES sessions?
  - *Indoor/Circuit*
  - *Walking session*
  - *Community delivered sessions*
- Can you tell me your thoughts about the themed discussion part of the SPACES sessions?
  - *Do you feel you learned new things about physical activity?*
- Can you tell me your thoughts about the social time of the SPACES sessions?
  - *Did you make new friends during the sessions/Have you stayed in touch with anyone you met during the SPACES sessions?*
- How did you feel about taking part in SPACES as part of a group?
  - *Was this different to other physical activity groups you’ve attended? If so, how?*
- How did you find the one-to-one appointments?
  - *How did they make you feel?*
  - *How useful did you find them?*
- How was your relationship with the Physical Activity Coordinators? During the weekly sessions? During the one-to-one appointments?
  - *Did you know [PAC] before you took part in SPACES?*
  - *How did the PACs make you feel?*
  - *What do you think makes a good PAC?*
- Is there any way we could improve the intervention?

***Intervention Drop-out questions***

- Can you tell me why you no longer felt able to take part in the sessions/intervention?
- Is there anything that would have made it possible for you to continue to take part in the sessions/intervention?

**Perceived benefits/problems:**

- Do you feel that you got what you were hoping to from the intervention?
  - *Did it match your expectations?*
  - *Do you think anything was missing?*
  - *Which parts were most/least useful?*
- Do you think taking part in the intervention has had an impact on your level of physical activity?
  - *How?*
  - *Have you continued to take part in physical activity since finishing the intervention?*
- How has the intervention affected your health and well-being, if at all (physical and mental)?
- Have your feelings about taking part in physical activity changed after taking part in the intervention?
  - *If so, how? Positively or negatively?*
- If you think back to the start of the intervention, what did you feel like the biggest obstacle for you to take part in physical activity?
  - *Has that changed since taking part in the SPACES intervention?*

Topic Guide for Physical Activity Coordinators

*Suggested questions are for guidance only. Use a semi-structured approach to explore ideas and views raised by participants where appropriate.*

**Introduction:** **Welcome and reminder of key points**

- Our interest is in your experiences of delivering the SPACES intervention, you are welcome to share anything you would like about this.
- There are no right or wrong answers
- Everything shared will remain confidential. The only time we may have to tell someone what you have said to us is if you say something which suggests there is a risk of significant harm to someone (including you).
- The conversation will be audio recorded but, once typed up, your responses will be anonymous and you will not be identified by name in any written reports. If participant happy to proceed turn on audio-recorder.

**What was your experience of delivering the SPACES intervention?**

*Optional prompts:*

**Accessibility:**

- Can you tell me your thoughts about the training you received for the SPACES intervention?
  - *Was anything missing?*
  - *Did you feel prepared to deliver the intervention following the training?*
- Can you tell me your thoughts about the PAC manual?
- What was your experience of the supervision sessions for PACs? (The PAC meetings)
  - *Did you find these useful?*
  - *How could these be improved?*
- Frequency – How did you feel about the number of sessions you delivered?
  - *Do you think that the number of one-to-one appointments with service users was appropriate? Or would you have liked more/less?*
- Timing/ Duration – How did you feel about the duration of the SPACES sessions?
  - *How did you feel about the timing of the sessions (time of day)?*
- Location – What are your thoughts on where the sessions were delivered?
  - *Appropriateness?*
  - *Accessibility?*
  - *Access to resources?*

**Acceptability:**

- Did you feel confident in delivering the SPACES intervention?
  - *Group sessions*
  - *One-to-one appointments*
- Did you feel sufficiently supported in delivering the SPACES intervention?
- Do you feel there are transferable skills between the SPACES intervention and your routine practice?
- Did you find any part of the intervention difficult, or did anything cause you concern?
- What do you feel participants found most valuable about the intervention? (activity, social time, discussions etc)
- Is there anything you would change to improve intervention delivery?
  - *Structure?*
  - *Types of activity*

**Interactions with participants:**

- How did you find delivering the intervention to participants?
  - *(e.g., explanation of information, how involved the participant was, how motivated they seemed to be)?*
- Can you describe any particular challenges in delivering group physical activity sessions to patients with severe mental ill health?
  - *Do you have any thoughts or experience about overcoming these challenges?*
