# Supplementary figures and images for: Better together: Service user and delivery staff experiences of the SPACES physical activity intervention for people with severe mental illness - a qualitative study of a feasibility trial
Source: Ment Health Phys Act. Author manuscript; Available in PMC 2025 Dec 4. (PMC7618440; doi:10.1016/j.mhpa.2025.100717)

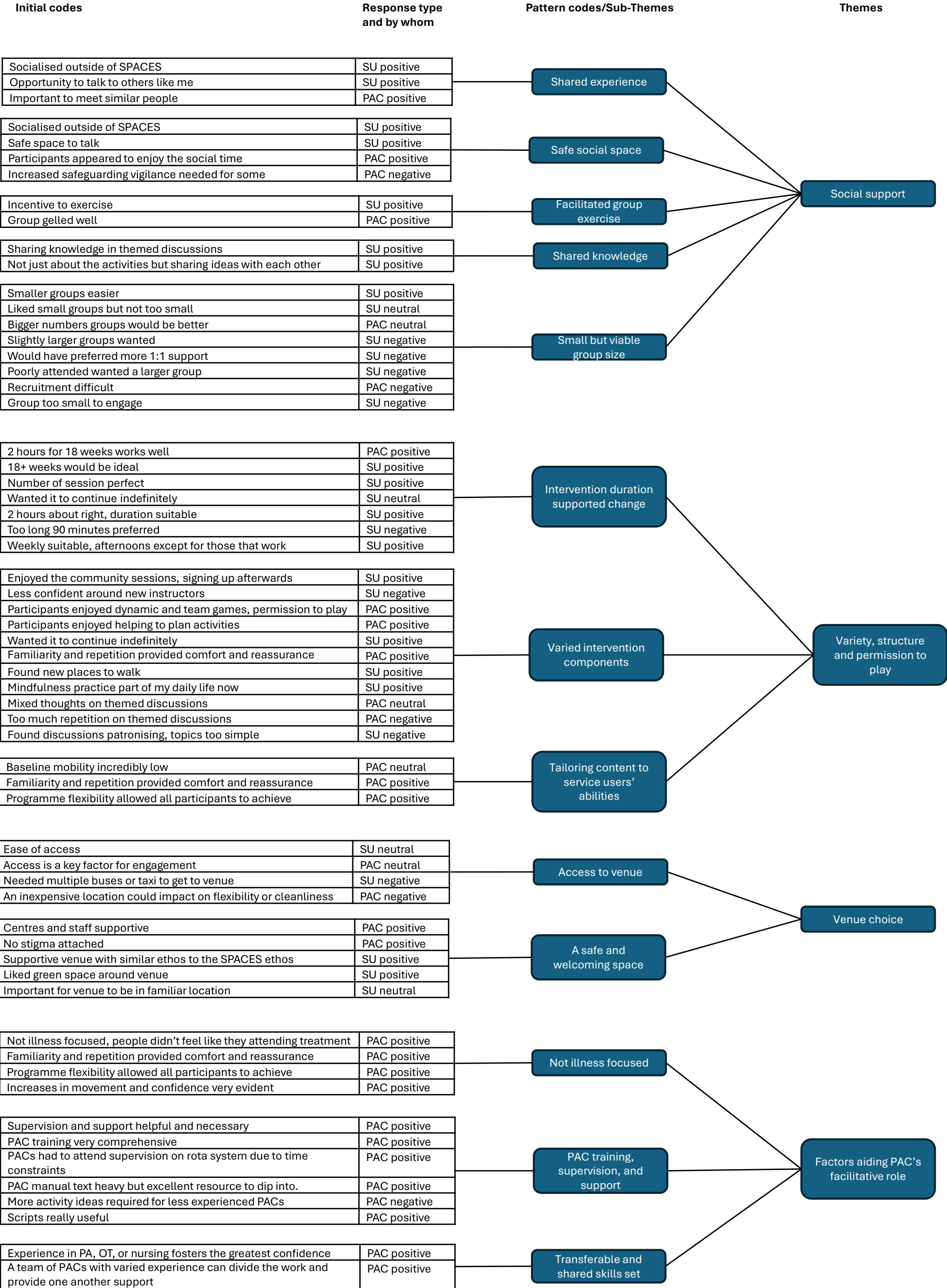

Supplement: Supplementary Material [file EMS210840-supplement-Supplementary_Material.zip › 1-s2.0-S1755296625000481-mmc4.pdf]
